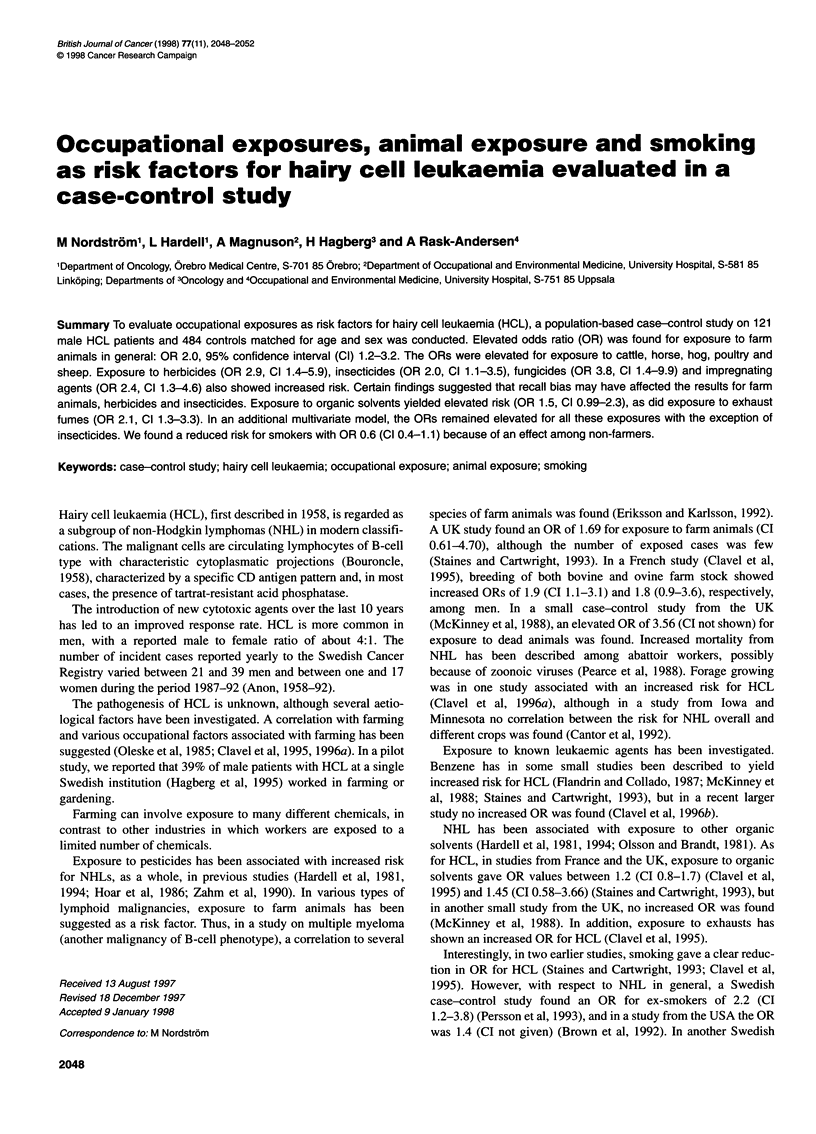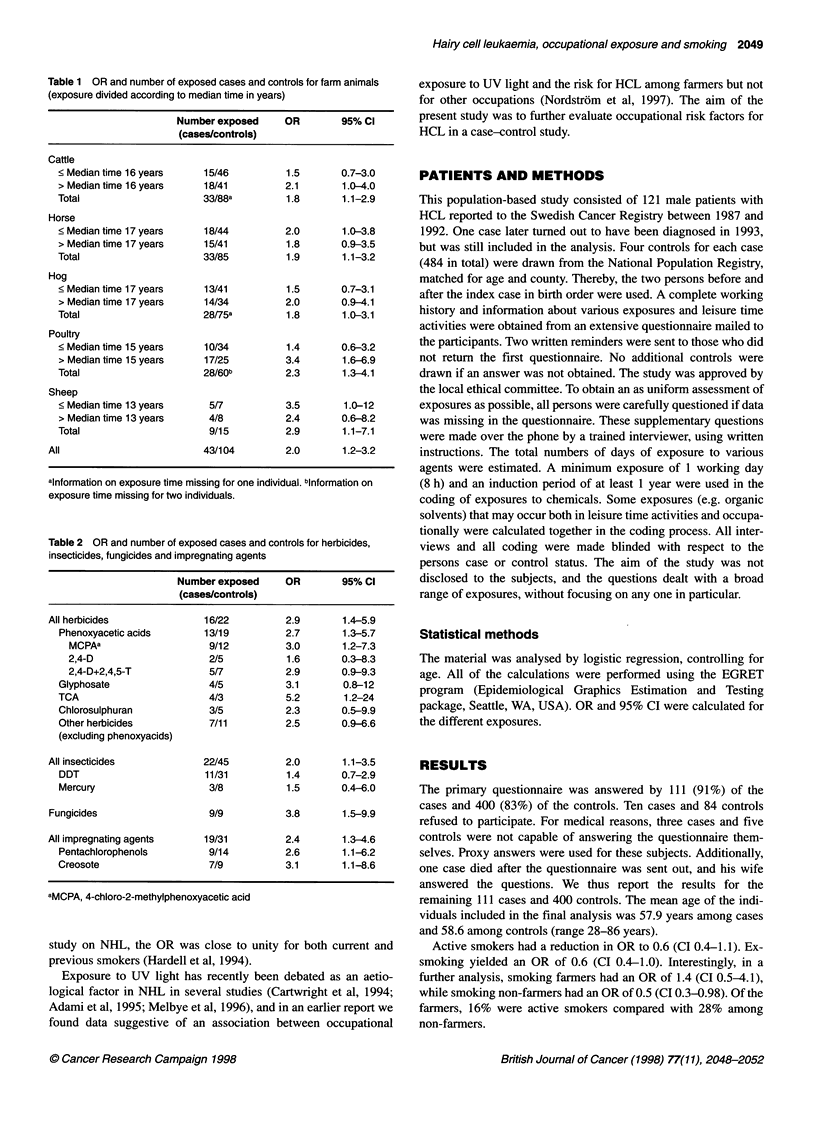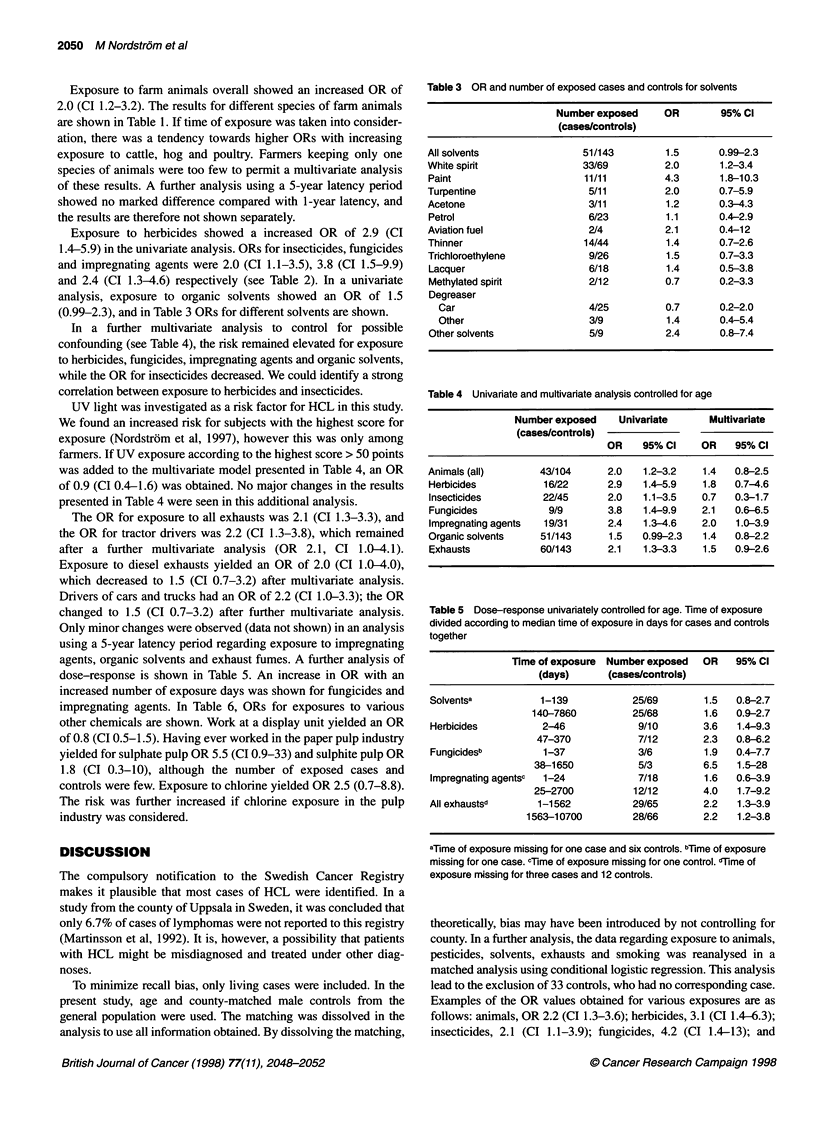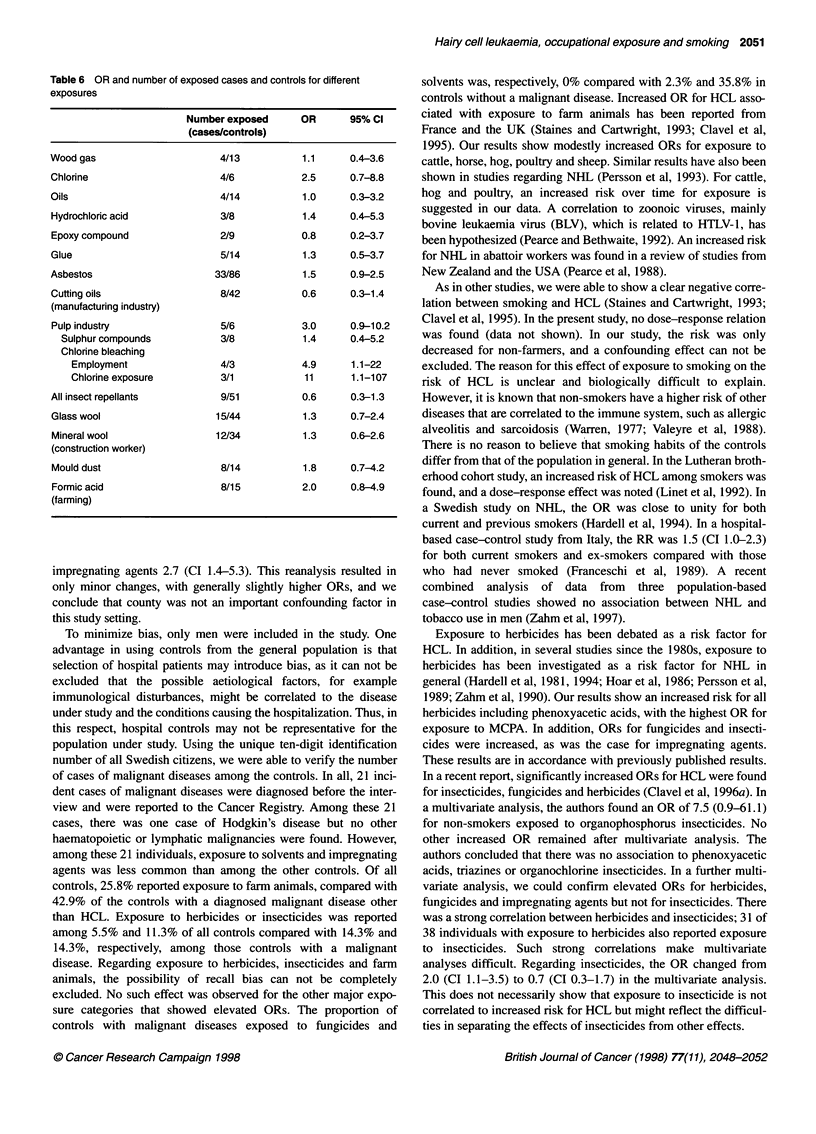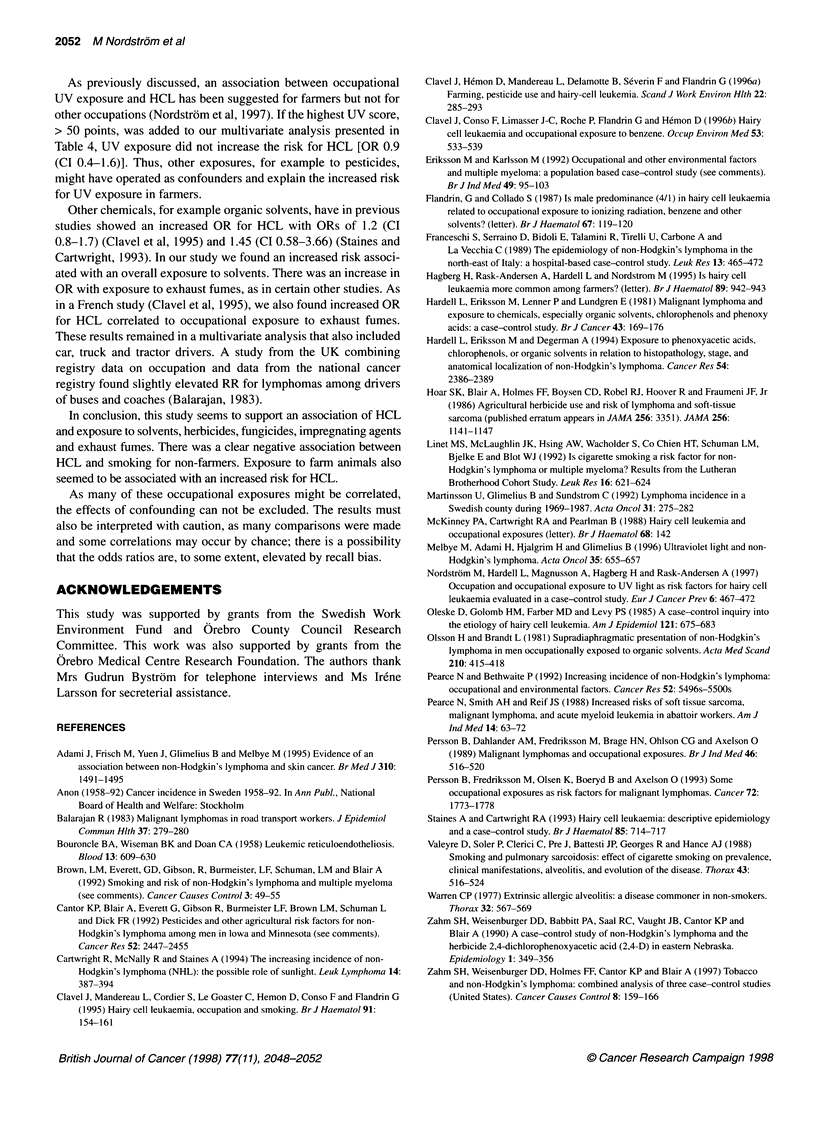# Occupational exposures, animal exposure and smoking as risk factors for hairy cell leukaemia evaluated in a case-control study.

**DOI:** 10.1038/bjc.1998.341

**Published:** 1998-06

**Authors:** M. NordstrÃ¶m, L. Hardell, A. Magnuson, H. Hagberg, A. Rask-Andersen

**Affiliations:** Department of Oncology, Orebro Medical Centre.

## Abstract

To evaluate occupational exposures as risk factors for hairy cell leukaemia (HCL), a population-based case-control study on 121 male HCL patients and 484 controls matched for age and sex was conducted. Elevated odds ratio (OR) was found for exposure to farm animals in general: OR 2.0, 95% confidence interval (CI) 1.2-3.2. The ORs were elevated for exposure to cattle, horse, hog, poultry and sheep. Exposure to herbicides (OR 2.9, CI 1.4-5.9), insecticides (OR 2.0, CI 1.1-3.5), fungicides (OR 3.8, CI 1.4-9.9) and impregnating agents (OR 2.4, CI 1.3-4.6) also showed increased risk. Certain findings suggested that recall bias may have affected the results for farm animals, herbicides and insecticides. Exposure to organic solvents yielded elevated risk (OR 1.5, CI 0.99-2.3), as did exposure to exhaust fumes (OR 2.1, CI 1.3-3.3). In an additional multivariate model, the ORs remained elevated for all these exposures with the exception of insecticides. We found a reduced risk for smokers with OR 0.6 (CI 0.4-1.1) because of an effect among non-farmers.


British Journal of Cancer (1998) 77(11), 2048–2052
© 1998 Cancer Research Campaign

### Occupational exposures, animal exposure and smoking as risk factors for hairy cell leukaemia evaluated in a case-control study

**M Nordström[1], L Hardell[1], A Magnuson[2], H Hagberg[3] and A Rask-Andersen[4]**

[1]Department of Oncology, Örebro Medical Centre, S-701 85 Örebro; [2]Department of Occupational and Environmental Medicine, University Hospital, S-581 85 Linköping; Departments of [3]Oncology and [4]Occupational and Environmental Medicine, University Hospital, S-751 85 Uppsala

**Summary** To evaluate occupational exposures as risk factors for hairy cell leukaemia (HCL), a population-based case–control study on 121 male HCL patients and 484 controls matched for age and sex was conducted. Elevated odds ratio (OR) was found for exposure to farm animals in general: OR 2.0, 95% confidence interval (CI) 1.2–3.2. The ORs were elevated for exposure to cattle, horse, hog, poultry and sheep. Exposure to herbicides (OR 2.9, CI 1.4–5.9), insecticides (OR 2.0, CI 1.1–3.5), fungicides (OR 3.8, CI 1.4–9.9) and impregnating agents (OR 2.4, CI 1.3–4.6) also showed increased risk. Certain findings suggested that recall bias may have affected the results for farm animals, herbicides and insecticides. Exposure to organic solvents yielded elevated risk (OR 1.5, CI 0.99–2.3), as did exposure to exhaust fumes (OR 2.1, CI 1.3–3.3). In an additional multivariate model, the ORs remained elevated for all these exposures with the exception of insecticides. We found a reduced risk for smokers with OR 0.6 (CI 0.4–1.1) because of an effect among non-farmers.

**Keywords:** case–control study; hairy cell leukaemia; occupational exposure; animal exposure; smoking

Hairy cell leukaemia (HCL), first described in 1958, is regarded as a subgroup of non-Hodgkin lymphomas (NHL) in modern classifications. The malignant cells are circulating lymphocytes of B-cell type with characteristic cytoplasmatic projections (Bouroncle, 1958), characterized by a specific CD antigen pattern and, in most cases, the presence of tartrat-resistant acid phosphatase.

The introduction of new cytotoxic agents over the last 10 years has led to an improved response rate. HCL is more common in men, with a reported male to female ratio of about 4:1. The number of incident cases reported yearly to the Swedish Cancer Registry varied between 21 and 39 men and between one and 17 women during the period 1987–92 (Anon, 1958–92).

The pathogenesis of HCL is unknown, although several aetiological factors have been investigated. A correlation with farming and various occupational factors associated with farming has been suggested (Oleske et al, 1985; Clavel et al, 1995, 1996*a*). In a pilot study, we reported that 39% of male patients with HCL at a single Swedish institution (Hagberg et al, 1995) worked in farming or gardening.

Farming can involve exposure to many different chemicals, in contrast to other industries in which workers are exposed to a limited number of chemicals.

Exposure to pesticides has been associated with increased risk for NHLs, as a whole, in previous studies (Hardell et al, 1981, 1994; Hoar et al, 1986; Zahm et al, 1990). In various types of lymphoid malignancies, exposure to farm animals has been suggested as a risk factor. Thus, in a study on multiple myeloma (another malignancy of B-cell phenotype), a correlation to several species of farm animals was found (Eriksson and Karlsson, 1992). A UK study found an OR of 1.69 for exposure to farm animals (CI 0.61–4.70), although the number of exposed cases was few (Staines and Cartwright, 1993). In a French study (Clavel et al, 1995), breeding of both bovine and ovine farm stock showed increased ORs of 1.9 (CI 1.1–3.1) and 1.8 (0.9–3.6), respectively, among men. In a small case–control study from the UK (McKinney et al, 1988), an elevated OR of 3.56 (CI not shown) for exposure to dead animals was found. Increased mortality from NHL has been described among abattoir workers, possibly because of zoonoic viruses (Pearce et al, 1988). Forage growing was in one study associated with an increased risk for HCL (Clavel et al, 1996*a*), although in a study from Iowa and Minnesota no correlation between the risk for NHL overall and different crops was found (Cantor et al, 1992).

Exposure to known leukaemic agents has been investigated. Benzene has in some small studies been described to yield increased risk for HCL (Flandrin and Collado, 1987; McKinney et al, 1988; Staines and Cartwright, 1993), but in a recent larger study no increased OR was found (Clavel et al, 1996*b*).

NHL has been associated with exposure to other organic solvents (Hardell et al, 1981, 1994; Olsson and Brandt, 1981). As for HCL, in studies from France and the UK, exposure to organic solvents gave OR values between 1.2 (CI 0.8–1.7) (Clavel et al, 1995) and 1.45 (CI 0.58–3.66) (Staines and Cartwright, 1993), but in another small study from the UK, no increased OR was found (McKinney et al, 1988). In addition, exposure to exhausts has shown an increased OR for HCL (Clavel et al, 1995).

Interestingly, in two earlier studies, smoking gave a clear reduction in OR for HCL (Staines and Cartwright, 1993; Clavel et al, 1995). However, with respect to NHL in general, a Swedish case–control study found an OR for ex-smokers of 2.2 (CI 1.2–3.8) (Persson et al, 1993), and in a study from the USA the OR was 1.4 (CI not given) (Brown et al, 1992). In another Swedish

*Received 13 August 1997*
*Revised 18 December 1997*
*Accepted 9 January 1998*

*Correspondence to:* M Nordström

**Table 1** OR and number of exposed cases and controls for farm animals (exposure divided according to median time in years)

| | Number exposed (cases/controls) | OR | 95% CI |
|---|---|---|---|
| Cattle | | | |
| ≤ Median time 16 years | 15/46 | 1.5 | 0.7–3.0 |
| > Median time 16 years | 18/41 | 2.1 | 1.0–4.0 |
| Total | 33/88[a] | 1.8 | 1.1–2.9 |
| Horse | | | |
| ≤ Median time 17 years | 18/44 | 2.0 | 1.0–3.8 |
| > Median time 17 years | 15/41 | 1.8 | 0.9–3.5 |
| Total | 33/85 | 1.9 | 1.1–3.2 |
| Hog | | | |
| ≤ Median time 17 years | 13/41 | 1.5 | 0.7–3.1 |
| > Median time 17 years | 14/34 | 2.0 | 0.9–4.1 |
| Total | 28/75[a] | 1.8 | 1.0–3.1 |
| Poultry | | | |
| ≤ Median time 15 years | 10/34 | 1.4 | 0.6–3.2 |
| > Median time 15 years | 17/25 | 3.4 | 1.6–6.9 |
| Total | 28/60[b] | 2.3 | 1.3–4.1 |
| Sheep | | | |
| ≤ Median time 13 years | 5/7 | 3.5 | 1.0–12 |
| > Median time 13 years | 4/8 | 2.4 | 0.6–8.2 |
| Total | 9/15 | 2.9 | 1.1–7.1 |
| All | 43/104 | 2.0 | 1.2–3.2 |

[a]Information on exposure time missing for one individual. [b]Information on exposure time missing for two individuals.

**Table 2** OR and number of exposed cases and controls for herbicides, insecticides, fungicides and impregnating agents

| | Number exposed (cases/controls) | OR | 95% CI |
|---|---|---|---|
| All herbicides | 16/22 | 2.9 | 1.4–5.9 |
| Phenoxyacetic acids | 13/19 | 2.7 | 1.3–5.7 |
| MCPA[a] | 9/12 | 3.0 | 1.2–7.3 |
| 2,4-D | 2/5 | 1.6 | 0.3–8.3 |
| 2,4-D+2,4,5-T | 5/7 | 2.9 | 0.9–9.3 |
| Glyphosate | 4/5 | 3.1 | 0.8–12 |
| TCA | 4/3 | 5.2 | 1.2–24 |
| Chlorosulphuran | 3/5 | 2.3 | 0.5–9.9 |
| Other herbicides (excluding phenoxyacids) | 7/11 | 2.5 | 0.9–6.6 |
| All insecticides | 22/45 | 2.0 | 1.1–3.5 |
| DDT | 11/31 | 1.4 | 0.7–2.9 |
| Mercury | 3/8 | 1.5 | 0.4–6.0 |
| Fungicides | 9/9 | 3.8 | 1.5–9.9 |
| All impregnating agents | 19/31 | 2.4 | 1.3–4.6 |
| Pentachlorophenols | 9/14 | 2.6 | 1.1–6.2 |
| Creosote | 7/9 | 3.1 | 1.1–8.6 |

[a]MCPA, 4-chloro-2-methylphenoxyacetic acid study on NHL, the OR was close to unity for both current and previous smokers (Hardell et al, 1994).

Exposure to UV light has recently been debated as an aetiological factor in NHL in several studies (Cartwright et al, 1994; Adami et al, 1995; Melbye et al, 1996), and in an earlier report we found data suggestive of an association between occupational exposure to UV light and the risk for HCL among farmers but not for other occupations (Nordström et al, 1997). The aim of the present study was to further evaluate occupational risk factors for HCL in a case–control study.

### PATIENTS AND METHODS

This population-based study consisted of 121 male patients with HCL reported to the Swedish Cancer Registry between 1987 and 1992. One case later turned out to have been diagnosed in 1993, but was still included in the analysis. Four controls for each case (484 in total) were drawn from the National Population Registry, matched for age and county. Thereby, the two persons before and after the index case in birth order were used. A complete working history and information about various exposures and leisure time activities were obtained from an extensive questionnaire mailed to the participants. Two written reminders were sent to those who did not return the first questionnaire. No additional controls were drawn if an answer was not obtained. The study was approved by the local ethical committee. To obtain an as uniform assessment of exposures as possible, all persons were carefully questioned if data was missing in the questionnaire. These supplementary questions were made over the phone by a trained interviewer, using written instructions. The total numbers of days of exposure to various agents were estimated. A minimum exposure of 1 working day (8 h) and an induction period of at least 1 year were used in the coding of exposures to chemicals. Some exposures (e.g. organic solvents) that may occur both in leisure time activities and occupationally were calculated together in the coding process. All interviews and all coding were made blinded with respect to the persons case or control status. The aim of the study was not disclosed to the subjects, and the questions dealt with a broad range of exposures, without focusing on any one in particular.

### Statistical methods

The material was analysed by logistic regression, controlling for age. All of the calculations were performed using the EGRET program (Epidemiological Graphics Estimation and Testing package, Seattle, WA, USA). OR and 95% CI were calculated for the different exposures.

### RESULTS

The primary questionnaire was answered by 111 (91%) of the cases and 400 (83%) of the controls. Ten cases and 84 controls refused to participate. For medical reasons, three cases and five controls were not capable of answering the questionnaire themselves. Proxy answers were used for these subjects. Additionally, one case died after the questionnaire was sent out, and his wife answered the questions. We thus report the results for the remaining 111 cases and 400 controls. The mean age of the individuals included in the final analysis was 57.9 years among cases and 58.6 among controls (range 28–86 years).

Active smokers had a reduction in OR to 0.6 (CI 0.4–1.1). Ex-smoking yielded an OR of 0.6 (CI 0.4–1.0). Interestingly, in a further analysis, smoking farmers had an OR of 1.4 (CI 0.5–4.1), while smoking non-farmers had an OR of 0.5 (CI 0.3–0.98). Of the farmers, 16% were active smokers compared with 28% among non-farmers.

© Cancer Research Campaign 1998 

Exposure to farm animals overall showed an increased OR of 2.0 (CI 1.2–3.2). The results for different species of farm animals are shown in Table 1. If time of exposure was taken into consideration, there was a tendency towards higher ORs with increasing exposure to cattle, hog and poultry. Farmers keeping only one species of animals were too few to permit a multivariate analysis of these results. A further analysis using a 5-year latency period showed no marked difference compared with 1-year latency, and the results are therefore not shown separately.

Exposure to herbicides showed a increased OR of 2.9 (CI 1.4–5.9) in the univariate analysis. ORs for insecticides, fungicides and impregnating agents were 2.0 (CI 1.1–3.5), 3.8 (CI 1.5–9.9) and 2.4 (CI 1.3–4.6) respectively (see Table 2). In a univariate analysis, exposure to organic solvents showed an OR of 1.5 (0.99–2.3), and in Table 3 ORs for different solvents are shown.

In a further multivariate analysis to control for possible confounding (see Table 4), the risk remained elevated for exposure to herbicides, fungicides, impregnating agents and organic solvents, while the OR for insecticides decreased. We could identify a strong correlation between exposure to herbicides and insecticides.

UV light was investigated as a risk factor for HCL in this study. We found an increased risk for subjects with the highest score for exposure (Nordström et al, 1997), however this was only among farmers. If UV exposure according to the highest score > 50 points was added to the multivariate model presented in Table 4, an OR of 0.9 (CI 0.4–1.6) was obtained. No major changes in the results presented in Table 4 were seen in this additional analysis.

The OR for exposure to all exhausts was 2.1 (CI 1.3–3.3), and the OR for tractor drivers was 2.2 (CI 1.3–3.8), which remained after a further multivariate analysis (OR 2.1, CI 1.0–4.1). Exposure to diesel exhausts yielded an OR of 2.0 (CI 1.0–4.0), which decreased to 1.5 (CI 0.7–3.2) after multivariate analysis. Drivers of cars and trucks had an OR of 2.2 (CI 1.0–3.3); the OR changed to 1.5 (CI 0.7–3.2) after further multivariate analysis. Only minor changes were observed (data not shown) in an analysis using a 5-year latency period regarding exposure to impregnating agents, organic solvents and exhaust fumes. A further analysis of dose–response is shown in Table 5. An increase in OR with an increased number of exposure days was shown for fungicides and impregnating agents. In Table 6, ORs for exposures to various other chemicals are shown. Work at a display unit yielded an OR of 0.8 (CI 0.5–1.5). Having ever worked in the paper pulp industry yielded for sulphate pulp OR 5.5 (CI 0.9–33) and sulphite pulp OR 1.8 (CI 0.3–10), although the number of exposed cases and controls were few. Exposure to chlorine yielded OR 2.5 (0.7–8.8). The risk was further increased if chlorine exposure in the pulp industry was considered.

**Table 3** OR and number of exposed cases and controls for solvents

| | Number exposed (cases/controls) | OR | 95% CI |
|---|---|---|---|
| All solvents | 51/143 | 1.5 | 0.99–2.3 |
| White spirit | 33/69 | 2.0 | 1.2–3.4 |
| Paint | 11/11 | 4.3 | 1.8–10.3 |
| Turpentine | 5/11 | 2.0 | 0.7–5.9 |
| Acetone | 3/11 | 1.2 | 0.3–4.3 |
| Petrol | 6/23 | 1.1 | 0.4–2.9 |
| Aviation fuel | 2/4 | 2.1 | 0.4–12 |
| Thinner | 14/44 | 1.4 | 0.7–2.6 |
| Trichloroethylene | 9/26 | 1.5 | 0.7–3.3 |
| Lacquer | 6/18 | 1.4 | 0.5–3.8 |
| Methylated spirit | 2/12 | 0.7 | 0.2–3.3 |
| Degreaser | | | |
| Car | 4/25 | 0.7 | 0.2–2.0 |
| Other | 3/9 | 1.4 | 0.4–5.4 |
| Other solvents | 5/9 | 2.4 | 0.8–7.4 |

**Table 4** Univariate and multivariate analysis controlled for age

| | Number exposed (cases/controls) | Univariate | | Multivariate | |
|---|---|---|---|---|---|
| | | OR | 95% CI | OR | 95% CI |
| Animals (all) | 43/104 | 2.0 | 1.2–3.2 | 1.4 | 0.8–2.5 |
| Herbicides | 16/22 | 2.9 | 1.4–5.9 | 1.8 | 0.7–4.6 |
| Insecticides | 22/45 | 2.0 | 1.1–3.5 | 0.7 | 0.3–1.7 |
| Fungicides | 9/9 | 3.8 | 1.4–9.9 | 2.1 | 0.6–6.5 |
| Impregnating agents | 19/31 | 2.4 | 1.3–4.6 | 2.0 | 1.0–3.9 |
| Organic solvents | 51/143 | 1.5 | 0.99–2.3 | 1.4 | 0.8–2.2 |
| Exhausts | 60/143 | 2.1 | 1.3–3.3 | 1.5 | 0.9–2.6 |

**Table 5** Dose–response univariately controlled for age. Time of exposure divided according to median time of exposure in days for cases and controls together

| | Time of exposure (days) | Number exposed (cases/controls) | OR | 95% CI |
|---|---|---|---|---|
| Solvents[a] | 1–139 | 25/69 | 1.5 | 0.8–2.7 |
| | 140–7860 | 25/68 | 1.6 | 0.9–2.7 |
| Herbicides | 2–46 | 9/10 | 3.6 | 1.4–9.3 |
| | 47–370 | 7/12 | 2.3 | 0.8–6.2 |
| Fungicides[b] | 1–37 | 3/6 | 1.9 | 0.4–7.7 |
| | 38–1650 | 5/3 | 6.5 | 1.5–28 |
| Impregnating agents[c] | 1–24 | 7/18 | 1.6 | 0.6–3.9 |
| | 25–2700 | 12/12 | 4.0 | 1.7–9.2 |
| All exhausts[d] | 1–1562 | 29/65 | 2.2 | 1.3–3.9 |
| | 1563–10700 | 28/66 | 2.2 | 1.2–3.8 |

[a]Time of exposure missing for one case and six controls. [b]Time of exposure missing for one case. [c]Time of exposure missing for one control. [d]Time of exposure missing for three cases and 12 controls.

### DISCUSSION

The compulsory notification to the Swedish Cancer Registry makes it plausible that most cases of HCL were identified. In a study from the county of Uppsala in Sweden, it was concluded that only 6.7% of cases of lymphomas were not reported to this registry (Martinsson et al, 1992). It is, however, a possibility that patients with HCL might be misdiagnosed and treated under other diagnoses.

To minimize recall bias, only living cases were included. In the present study, age and county-matched male controls from the general population were used. The matching was dissolved in the analysis to use all information obtained. By dissolving the matching, theoretically, bias may have been introduced by not controlling for county. In a further analysis, the data regarding exposure to animals, pesticides, solvents, exhausts and smoking was reanalysed in a matched analysis using conditional logistic regression. This analysis lead to the exclusion of 33 controls, who had no corresponding case. Examples of the OR values obtained for various exposures are as follows: animals, OR 2.2 (CI 1.3–3.6); herbicides, 3.1 (CI 1.4–6.3); insecticides, 2.1 (CI 1.1–3.9); fungicides, 4.2 (CI 1.4–13); and

*© Cancer Research Campaign 1998*

**Table 6** OR and number of exposed cases and controls for different exposures

| | Number exposed (cases/controls) | OR | 95% CI |
|---|---|---|---|
| Wood gas | 4/13 | 1.1 | 0.4–3.6 |
| Chlorine | 4/6 | 2.5 | 0.7–8.8 |
| Oils | 4/14 | 1.0 | 0.3–3.2 |
| Hydrochloric acid | 3/8 | 1.4 | 0.4–5.3 |
| Epoxy compound | 2/9 | 0.8 | 0.2–3.7 |
| Glue | 5/14 | 1.3 | 0.5–3.7 |
| Asbestos | 33/86 | 1.5 | 0.9–2.5 |
| Cutting oils (manufacturing industry) | 8/42 | 0.6 | 0.3–1.4 |
| Pulp industry | 5/6 | 3.0 | 0.9–10.2 |
| Sulphur compounds | 3/8 | 1.4 | 0.4–5.2 |
| Chlorine bleaching | | | |
| Employment | 4/3 | 4.9 | 1.1–22 |
| Chlorine exposure | 3/1 | 11 | 1.1–107 |
| All insect repellants | 9/51 | 0.6 | 0.3–1.3 |
| Glass wool | 15/44 | 1.3 | 0.7–2.4 |
| Mineral wool (construction worker) | 12/34 | 1.3 | 0.6–2.6 |
| Mould dust | 8/14 | 1.8 | 0.7–4.2 |
| Formic acid (farming) | 8/15 | 2.0 | 0.8–4.9 |

impregnating agents 2.7 (CI 1.4–5.3). This reanalysis resulted in only minor changes, with generally slightly higher ORs, and we conclude that county was not an important confounding factor in this study setting.

To minimize bias, only men were included in the study. One advantage in using controls from the general population is that selection of hospital patients may introduce bias, as it can not be excluded that the possible aetiological factors, for example immunological disturbances, might be correlated to the disease under study and the conditions causing the hospitalization. Thus, in this respect, hospital controls may not be representative for the population under study. Using the unique ten-digit identification number of all Swedish citizens, we were able to verify the number of cases of malignant diseases among the controls. In all, 21 incident cases of malignant diseases were diagnosed before the interview and were reported to the Cancer Registry. Among these 21 cases, there was one case of Hodgkin's disease but no other haematopoietic or lymphatic malignancies were found. However, among these 21 individuals, exposure to solvents and impregnating agents was less common than among the other controls. Of all controls, 25.8% reported exposure to farm animals, compared with 42.9% of the controls with a diagnosed malignant disease other than HCL. Exposure to herbicides or insecticides was reported among 5.5% and 11.3% of all controls compared with 14.3% and 14.3%, respectively, among those controls with a malignant disease. Regarding exposure to herbicides, insecticides and farm animals, the possibility of recall bias can not be completely excluded. No such effect was observed for the other major exposure categories that showed elevated ORs. The proportion of controls with malignant diseases exposed to fungicides and solvents was, respectively, 0% compared with 2.3% and 35.8% in controls without a malignant disease. Increased OR for HCL associated with exposure to farm animals has been reported from France and the UK (Staines and Cartwright, 1993; Clavel et al, 1995). Our results show modestly increased ORs for exposure to cattle, horse, hog, poultry and sheep. Similar results have also been shown in studies regarding NHL (Persson et al, 1993). For cattle, hog and poultry, an increased risk over time for exposure is suggested in our data. A correlation to zoonoic viruses, mainly bovine leukaemia virus (BLV), which is related to HTLV-1, has been hypothesized (Pearce and Bethwaite, 1992). An increased risk for NHL in abattoir workers was found in a review of studies from New Zealand and the USA (Pearce et al, 1988).

As in other studies, we were able to show a clear negative correlation between smoking and HCL (Staines and Cartwright, 1993; Clavel et al, 1995). In the present study, no dose–response relation was found (data not shown). In our study, the risk was only decreased for non-farmers, and a confounding effect can not be excluded. The reason for this effect of exposure to smoking on the risk of HCL is unclear and biologically difficult to explain. However, it is known that non-smokers have a higher risk of other diseases that are correlated to the immune system, such as allergic alveolitis and sarcoidosis (Warren, 1977; Valeyre et al, 1988). There is no reason to believe that smoking habits of the controls differ from that of the population in general. In the Lutheran brotherhood cohort study, an increased risk of HCL among smokers was found, and a dose–response effect was noted (Linet et al, 1992). In a Swedish study on NHL, the OR was close to unity for both current and previous smokers (Hardell et al, 1994). In a hospital-based case–control study from Italy, the RR was 1.5 (CI 1.0–2.3) for both current smokers and ex-smokers compared with those who had never smoked (Franceschi et al, 1989). A recent combined analysis of data from three population-based case–control studies showed no association between NHL and tobacco use in men (Zahm et al, 1997).

Exposure to herbicides has been debated as a risk factor for HCL. In addition, in several studies since the 1980s, exposure to herbicides has been investigated as a risk factor for NHL in general (Hardell et al, 1981, 1994; Hoar et al, 1986; Persson et al, 1989; Zahm et al, 1990). Our results show an increased risk for all herbicides including phenoxyacetic acids, with the highest OR for exposure to MCPA. In addition, ORs for fungicides and insecticides were increased, as was the case for impregnating agents. These results are in accordance with previously published results. In a recent report, significantly increased ORs for HCL were found for insecticides, fungicides and herbicides (Clavel et al, 1996*a*). In a multivariate analysis, the authors found an OR of 7.5 (0.9–61.1) for non-smokers exposed to organophosphorus insecticides. No other increased OR remained after multivariate analysis. The authors concluded that there was no association to phenoxyacetic acids, triazines or organochlorine insecticides. In a further multivariate analysis, we could confirm elevated ORs for herbicides, fungicides and impregnating agents but not for insecticides. There was a strong correlation between herbicides and insecticides; 31 of 38 individuals with exposure to herbicides also reported exposure to insecticides. Such strong correlations make multivariate analyses difficult. Regarding insecticides, the OR changed from 2.0 (CI 1.1–3.5) to 0.7 (CI 0.3–1.7) in the multivariate analysis. This does not necessarily show that exposure to insecticide is not correlated to increased risk for HCL but might reflect the difficulties in separating the effects of insecticides from other effects.

© Cancer Research Campaign 1998

As previously discussed, an association between occupational UV exposure and HCL has been suggested for farmers but not for other occupations (Nordström et al, 1997). If the highest UV score, > 50 points, was added to our multivariate analysis presented in Table 4, UV exposure did not increase the risk for HCL [OR 0.9 (CI 0.4–1.6)]. Thus, other exposures, for example to pesticides, might have operated as confounders and explain the increased risk for UV exposure in farmers.

Other chemicals, for example organic solvents, have in previous studies showed an increased OR for HCL with ORs of 1.2 (CI 0.8–1.7) (Clavel et al, 1995) and 1.45 (CI 0.58–3.66) (Staines and Cartwright, 1993). In our study we found an increased risk associated with an overall exposure to solvents. There was an increase in OR with exposure to exhaust fumes, as in certain other studies. As in a French study (Clavel et al, 1995), we also found increased OR for HCL correlated to occupational exposure to exhaust fumes. These results remained in a multivariate analysis that also included car, truck and tractor drivers. A study from the UK combining registry data on occupation and data from the national cancer registry found slightly elevated RR for lymphomas among drivers of buses and coaches (Balarajan, 1983).

In conclusion, this study seems to support an association of HCL and exposure to solvents, herbicides, fungicides, impregnating agents and exhaust fumes. There was a clear negative association between HCL and smoking for non-farmers. Exposure to farm animals also seemed to be associated with an increased risk for HCL.

As many of these occupational exposures might be correlated, the effects of confounding can not be excluded. The results must also be interpreted with caution, as many comparisons were made and some correlations may occur by chance; there is a possibility that the odds ratios are, to some extent, elevated by recall bias.

### ACKNOWLEDGEMENTS

This study was supported by grants from the Swedish Work Environment Fund and Örebro County Council Research Committee. This work was also supported by grants from the Örebro Medical Centre Research Foundation. The authors thank Mrs Gudrun Byström for telephone interviews and Ms Iréne Larsson for secreterial assistance.

### REFERENCES

Adami J, Frisch M, Yuen J, Glimelius B and Melbye M (1995) Evidence of an association between non-Hodgkin's lymphoma and skin cancer. *Br Med J* **310**: 1491–1495

Anon (1958–92) Cancer incidence in Sweden 1958–92. In *Ann Publ.*, National Board of Health and Welfare: Stockholm

Balarajan R (1983) Malignant lymphomas in road transport workers. *J Epidemiol Commun Hlth* **37**: 279–280

Bouroncle BA, Wiseman BK and Doan CA (1958) Leukemic reticuloendotheliosis. *Blood* **13**: 609–630

Brown, LM, Everett, GD, Gibson, R, Burmeister, LF, Schuman, LM and Blair A (1992) Smoking and risk of non-Hodgkin's lymphoma and multiple myeloma (see comments). *Cancer Causes Control* **3**: 49–55

Cantor KP, Blair A, Everett G, Gibson R, Burmeister LF, Brown LM, Schuman L and Dick FR (1992) Pesticides and other agricultural risk factors for non-Hodgkin's lymphoma among men in Iowa and Minnesota (see comments). *Cancer Res* **52**: 2447–2455

Cartwright R, McNally R and Staines A (1994) The increasing incidence of non-Hodgkin's lymphoma (NHL): the possible role of sunlight. *Leuk Lymphoma* **14**: 387–394

Clavel J, Mandereau L, Cordier S, Le Goaster C, Hemon D, Conso F and Flandrin G (1995) Hairy cell leukaemia, occupation and smoking. *Br J Haematol* **91**: 154–161

Clavel J, Hémon D, Mandereau L, Delamotte B, Séverin F and Flandrin G (1996*a*) Farming, pesticide use and hairy-cell leukemia. *Scand J Work Environ Hlth* **22**: 285–293

Clavel J, Conso F, Limasser J-C, Roche P, Flandrin G and Hémon D (1996*b*) Hairy cell leukaemia and occupational exposure to benzene. *Occup Environ Med* **53**: 533–539

Eriksson M and Karlsson M (1992) Occupational and other environmental factors and multiple myeloma: a population based case–control study (see comments). *Br J Ind Med* **49**: 95–103

Flandrin, G and Collado S (1987) Is male predominance (4/1) in hairy cell leukaemia related to occupational exposure to ionizing radiation, benzene and other solvents? (letter). *Br J Haematol* **67**: 119–120

Franceschi S, Serraino D, Bidoli E, Talamini R, Tirelli U, Carbone A and La Vecchia C (1989) The epidemiology of non-Hodgkin's lymphoma in the north-east of Italy: a hospital-based case–control study. *Leuk Res* **13**: 465–472

Hagberg H, Rask-Andersen A, Hardell L and Nordstrom M (1995) Is hairy cell leukaemia more common among farmers? (letter). *Br J Haematol* **89**: 942–943

Hardell L, Eriksson M, Lenner P and Lundgren E (1981) Malignant lymphoma and exposure to chemicals, especially organic solvents, chlorophenols and phenoxy acids: a case–control study. *Br J Cancer* **43**: 169–176

Hardell L, Eriksson M and Degerman A (1994) Exposure to phenoxyacetic acids, chlorophenols, or organic solvents in relation to histopathology, stage, and anatomical localization of non-Hodgkin's lymphoma. *Cancer Res* **54**: 2386–2389

Hoar SK, Blair A, Holmes FF, Boysen CD, Robel RJ, Hoover R and Fraumeni JF, Jr (1986) Agricultural herbicide use and risk of lymphoma and soft-tissue sarcoma (published erratum appears in *JAMA* **256**: 3351). *JAMA* **256**: 1141–1147

Linet MS, McLaughlin JK, Hsing AW, Wacholder S, Co Chien HT, Schuman LM, Bjelke E and Blot WJ (1992) Is cigarette smoking a risk factor for non-Hodgkin's lymphoma or multiple myeloma? Results from the Lutheran Brotherhood Cohort Study. *Leuk Res* **16**: 621–624

Martinsson U, Glimelius B and Sundstrom C (1992) Lymphoma incidence in a Swedish county during 1969–1987. *Acta Oncol* **31**: 275–282

McKinney PA, Cartwright RA and Pearlman B (1988) Hairy cell leukemia and occupational exposures (letter). *Br J Haematol* **68**: 142

Melbye M, Adami H, Hjalgrim H and Glimelius B (1996) Ultraviolet light and non-Hodgkin's lymphoma. *Acta Oncol* **35**: 655–657

Nordström M, Hardell L, Magnusson A, Hagberg H and Rask-Andersen A (1997) Occupation and occupational exposure to UV light as risk factors for hairy cell leukaemia evaluated in a case–control study. *Eur J Cancer Prev* **6**: 467–472

Oleske D, Golomb HM, Farber MD and Levy PS (1985) A case–control inquiry into the etiology of hairy cell leukemia. *Am J Epidemiol* **121**: 675–683

Olsson H and Brandt L (1981) Supradiaphragmatic presentation of non-Hodgkin's lymphoma in men occupationally exposed to organic solvents. *Acta Med Scand* **210**: 415–418

Pearce N and Bethwaite P (1992) Increasing incidence of non-Hodgkin's lymphoma: occupational and environmental factors. *Cancer Res* **52**: 5496s–5500s

Pearce N, Smith AH and Reif JS (1988) Increased risks of soft tissue sarcoma, malignant lymphoma, and acute myeloid leukemia in abattoir workers. *Am J Ind Med* **14**: 63–72

Persson B, Dahlander AM, Fredriksson M, Brage HN, Ohlson CG and Axelson O (1989) Malignant lymphomas and occupational exposures. *Br J Ind Med* **46**: 516–520

Persson B, Fredriksson M, Olsen K, Boeryd B and Axelson O (1993) Some occupational exposures as risk factors for malignant lymphomas. *Cancer* **72**: 1773–1778

Staines A and Cartwright RA (1993) Hairy cell leukaemia: descriptive epidemiology and a case–control study. *Br J Haematol* **85**: 714–717

Valeyre D, Soler P, Clerici C, Pre J, Battesti JP, Georges R and Hance AJ (1988) Smoking and pulmonary sarcoidosis: effect of cigarette smoking on prevalence, clinical manifestations, alveolitis, and evolution of the disease. *Thorax* **43**: 516–524

Warren CP (1977) Extrinsic allergic alveolitis: a disease commoner in non-smokers. *Thorax* **32**: 567–569

Zahm SH, Weisenburger DD, Babbitt PA, Saal RC, Vaught JB, Cantor KP and Blair A (1990) A case–control study of non-Hodgkin's lymphoma and the herbicide 2,4-dichlorophenoxyacetic acid (2,4-D) in eastern Nebraska. *Epidemiology* **1**: 349–356

Zahm SH, Weisenburger DD, Holmes FF, Cantor KP and Blair A (1997) Tobacco and non-Hodgkin's lymphoma: combined analysis of three case–control studies (United States). *Cancer Causes Control* **8**: 159–166

© Cancer Research Campaign 1998